# Antimicrobial Drug Administration and Antimicrobial Resistance of *Salmonella* Isolates Originating from the Broiler Production Value Chain in Nigeria

**DOI:** 10.3390/antibiotics8020075

**Published:** 2019-06-06

**Authors:** Nurudeen Olalekan Oloso, Ismail Adewuyi Adeyemo, Henriette van Heerden, Olubunmi Gabriel Fasanmi, Folorunso Oludayo Fasina

**Affiliations:** 1 Department of Production Animal Studies, Faculty of Veterinary Science, University of Pretoria, Onderstepoort 0110, South Africa; bumaetal@gmail.com; 2 Department of Veterinary Microbiology, Faculty of Veterinary Medicine, University of Ibadan, Ibadan 200284, Nigeria; adeyemo_ismail@yahoo.com; 3 Department of Veterinary Tropical Diseases, Faculty of Veterinary Science, University of Pretoria, Onderstepoort 0110, South Africa; Henriette.VanHeerden@up.ac.za; 4 Federal Colleges of Animal Health and Production Technology, Ibadan 200262, Nigeria; 5 Emergency Centre for Transboundary Animal Diseases—Food and Agriculture Organisation (ECTAD-FAO), Dar es Salaam 14111, United Republic of Tanzania

**Keywords:** food animal residue, antimicrobial usage, antimicrobial stewardship, environment, live bird market, breeder broiler, hatchery

## Abstract

*Salmonella* is among the pathogens on the high global priority lists for monitoring for studies on the discovery of new antimicrobials and understanding of how antimicrobial resistance (AMR) develops. AMR in connection with antibiotic usage patterns has been considered as a strong factor and contributor to the AMR pool. The purposes of use, pattern of antimicrobial drug administration, as well as the prevalence of AMR in *Salmonella* originating from the Nigeria broiler production value chain (NBPVC) was explored. A well-structured questionnaire on antimicrobial usage (n = 181) was used for sampling that focused on 21 antimicrobials from 151 locations. Simultaneously, AMR testing for 18 commonly used antimicrobials on *Salmonella* in humans was also carried out. Antimicrobial resistance Salmonella spp. were isolated in 23% of the samples (261 of 1135 samples from the broiler input, products, and the environment) using modified ISO 6579 and *invA* PCR protocols. Over 80% of the antimicrobials used in the NBPVC were administered without a veterinarian prescription. Prevalence of antimicrobial administration without prescription were as follows: live-bird-market (100%), hatchery (86.7%), grow-out-farm (75%), and breeder (66.7%). Widespread prophylactic and metaphylactic use of antimicrobials were recorded with the highest use seen for enrofloxacin (63% and 24%), tetracycline (58% and 33%), and erythromycin (50% and 17%). Antimicrobial resistance was highest for flumequine (100%), penicillin (95%), and perfloxacin (89%). High levels of use without laboratory support of a newer generation of a class of antibiotics suspected to confer high resistance on older generations of the same class (quinolones) was observed.

## 1. Introduction

The advent of antimicrobials and their use for the treatment of infections in human and animal health remains a turning point in the field of therapeutics, and this discovery and success has been followed by its use for other purposes, such as growth promotion [1,2]. These alternative uses are borne out of economic interest, poor farm management practices, and the push for large scale production [1,2,3]. Paradoxically, microbial organisms have responded through many phenotypic or genotypic evolutionary processes in an attempt to adapt and survive, a phenomenon referred to as antimicrobial resistance (AMR) [2,3]. AMR is the ability of a microorganism (bacteria, viruses, and certain parasites) to prevent an antimicrobial agent (antibiotics, antivirals, and antimalarials) from its predetermined level of effectiveness against such microorganisms [3]. Earlier inventions and production of new drugs have led to the generation of several classes of new antimicrobials [2]. However, the announcements of such milestones in drug discovery have been followed by reports of resistance development by some organisms in the short to medium term [4,5]. Meanwhile, the process of drug development often requires great resources and faces enormous challenges associated with clinical trials, a prerequisite before an acceptance of any drug for usage [4,5]. This issue has greatly contributed to reducing the rate of invention of new antimicrobials over the last decades [5]. This trend in a reduced rate of drug development and over-dependence on antimicrobials in public health and veterinary medicine continues at epidemic proportions, particularly in the developing countries, where standardization and good farming practices are often not feasible [3]. The magnitude of AMR threats received the highest level of political commitment from world leaders at the United Nation General Assembly in 2016, and a political declaration on antimicrobials was issued [6]. The World Health Organization (WHO) had also issued a list of antimicrobial resistant bacteria that required priority attention in order to guide research, discoveries, and the development of new antimicrobials. These studies will aim at finding empirical methods of treatment and control in confronting AMR threats [7]. *Salmonella* was one such organism that have been listed on the high Global Priority list of the WHO [7]. 

Since AMR is a complex and persistent threat in global health management, surveillance for and careful monitoring for AMR has therefore become an important component of an overall strategy to institute good antimicrobial stewardship [5]. Food producing animals, including poultry, have been identified as major contributors to the current AMR pool in the human–animal–environment tripod [3]. Poultry serves as an important animal protein and food source in Nigeria, and a recent upsurge in broiler production is associated with increased consumption of poultry in Nigeria. Broiler has a fast turnover in terms of production, and it is a widely accepted food that has the capacity to meet the needs of human population growth [8,9]. Although surveillance for zoonotic food borne pathogens has been identified as important, its coordination to tackle antimicrobial resistance in Africa remains suspect [3]. In addition, carefully instituted protocols and a system of good antimicrobial stewardship that covers human, food animals, and the shared environments are lacking [9]. In Nigeria, low levels of compliance with biosecurity practices, especially in the Nigeria broiler production value chain (NBPVC) is intricately linked with poor husbandry practices, thus necessitating a reliance and over-dependence on antimicrobials for animal production purposes [3,8,9]. 

The Nigeria Centre for Disease Control (NCDC) in collaboration with relevant national institutions and other stakeholders have reviewed grey literature and peer-reviewed reports on several food-borne pathogens and established the situation of AMR in humans, food animals, and their shared environment in Nigeria [3]. Observation of high levels of AMR in humans, food animals, including poultry, and the environment have also been reported in studies from parts of Africa, including Nigeria [3,10]. In light of this, there is a need for a carefully planned intensive surveillance for AMR in all countries as recommended by the WHO [3,4,5,10], with a particular focus of attention on areas where information is currently scarce [3]. 

Previous studies on AMR in Nigeria poultry have concentrated more on the commercial laying flock and their products, particularly eggs, hence the dearth of information and data on AMR in the Nigeria broiler production value chain [3]. The structure of the NBPVC has been elaborated in other studies in order to understand the industry with a view to gaining insight into where antimicrobial abuse may be occurring and where interventions are needed [11]. Six critical stages have been identified in the structure of the NBPVC [11]: (1) Broiler Breeder Farms (BF) consist of breeding flocks of the grandparent or parent stock; (2) Hatcheries are sections or separate companies where the presumed fertile eggs from the BF are processed to broiler day-old chicks (DOC); (3) Grow-out Farms are the stage where the DOCs are raised to be table-size birds; (4) Abattoirs are the stage where the table size broilers are processed and packaged as fresh or frozen broiler bird products; (5) Retail Outlets are sections or separate companies where the broiler bird products are sold as fresh or frozen to consumers; (6) Live Bird Markets (LBM) are places where birds are sold live to customers [11]. This study explored the dynamics and patterns of antimicrobial drug administration in the NBPVC and evaluated the antimicrobial resistance of *Salmonella* isolates originating therefrom. Information generated thereof should serve as baseline data for subsequent studies and create links between antimicrobial usage and stewardship, broilers, *Salmonella*, and antimicrobial resistance in the Nigerian poultry. 

## 2. Materials and Methods

### 2.1. Study Design

**Figure 1:**
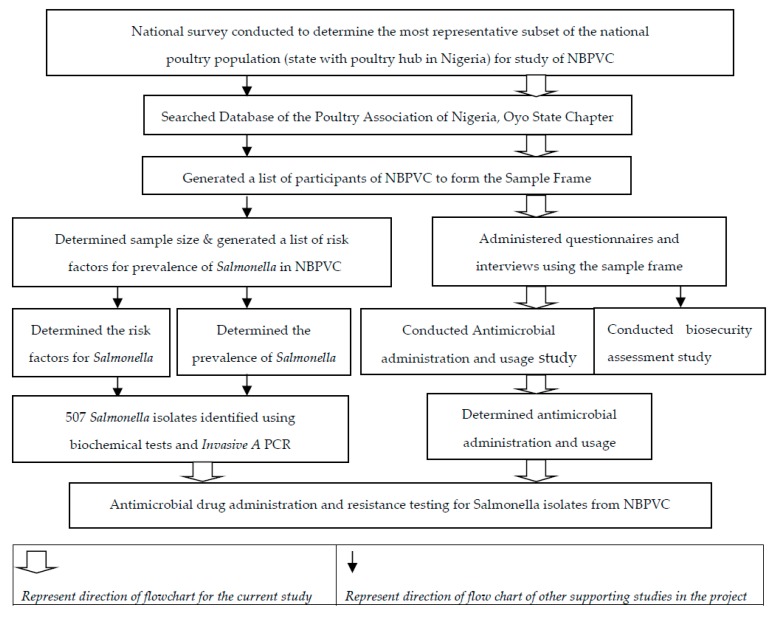
Schematic diagram of the study of antimicrobial susceptibility testing of *Salmonella* isolates and antimicrobial drug administration in the broiler production value chain in Nigeria (NBPVC).

The flowchart below details the sequence of the study (Figure 1). First, through a national survey, Oyo State, South West Nigeria was chosen as the study area. 

A national survey was conducted among 348 participants listed by interest groups who attended the 2016 joint congress of the African Veterinary Association & Nigerian Veterinary Medical Association (AVA/NVMA). The objective of the national survey was to obtain general information concerning the poultry production system and value chain and to select the state that would be the best representative as a case study for the Nigeria broiler production value chain (NBPVC). Oyo State was selected as a preferred representative poultry production hub for Nigeria (based on consensus of 76% of all respondents) with reasons including the presence of the only central open day-old chicks market in the country, a central point for several stakeholders; the highest number of head offices or presence of industrial poultry companies; the highest number of hatcheries; and the highest number of poultry abattoirs. Also, two out of the four companies licensed by the Federal Republic of Nigeria (FRN) to import grandparent stock (GS) have their GS farms in the state. Between September 2016 and September 2017, members of the Poultry Association of Nigeria (PAN), Oyo state, and the NVMA, Oyo state branch were interviewed (*n* = 464). 

Follow-up visits were made to the open day-old chicks market, farm, hatchery, abattoir, and other locations relevant to the study. Oyo state, Nigeria is located between geographical coordinates 8.1196° N, 3.4196° E [11]. Based on the information obtained, the following six stages of the value chain were identified as the most critical in the NBPVC: breeder, hatchery, grow-out, abattoir, retail, and live-bird-market [8,11]. Antimicrobial drug administration and usage surveys were conducted in these selected stages, and based on the information obtained and the list of critical and important drugs in animals outlined by the World Organization for Animal Health (OIE) [12], a total of 21 antimicrobial drugs were identified as commonly used in the NBPVC. Of these 21 drugs, three were peculiarly used only in animals. The remaining 18 are among the commonly used drugs in humans and listed by the WHO as critically important in human medicine for health management. These drugs were selected for the antimicrobial susceptibility testing (AST) study [12,13,14]. 

Concurrently with the survey, biological samples were collected in order to determine the prevalence, antimicrobial resistance, serotyping, and molecular characterization of *Salmonella* from these selected sites [11,14]; in addition, an AST study was performed on *Salmonella* isolated thereof (Figure 1, Supplementary Materials Figures S1 and S2). Specifically, the purpose(s) for antimicrobial drug administration and assessment of *Salmonella* isolates resistance patterns were conducted as outlined below (Figure 1; Supplementary Materials Figures S1 and S2) [11]. The *Salmonella* isolates in this study were not identified to species level after the identification with the *invA* gene PCR (Supplementary Materials Figure S2). 

### 2.2. Antimicrobial Drug Administration and Usage Survey

Between August 2016 and April 2017, a total of 151 sampling sites (Table 1) were selected from the list supplied by PAN, Oyo State, for the questionnaire survey on antimicrobial drug use [11]. Inclusion criteria was for a selected site to have been active in broiler production for at least three years. The questionnaire was aimed at investigating the records of private drug inventories/administration records of broiler breeder farms (BF, *n* = 45), hatcheries (H, *n* = 15), grow-out farms (GF, *n* = 76), and live bird markets (LBM, *n* = 45 (3 per LBM X 15 LBMs)). The abattoir and retail markets were excluded from the survey at this stage of the NBPVC exploration (Figure 1, Table 1). Each questionnaire was treated as a discrete unit representing only one poultry population, and each antimicrobial was referred to by its generic name irrespective of its brand or proprietary name. 

The information obtained was categorized into four intents of usage of antimicrobial based on the standard specification of the Food Animal Residue Avoidance Databank (FARAD) [15]. This includes: (1) Prophylaxis: where it is incorporated into feed or water regularly as an additive or supplement without any link to any form of required treatment or where the antibiotic is listed as a component of a drug consisting of small doses, (low) concentration, of many drugs below the standard recommendation; (2) Metaphylaxis or therapeutics without laboratory tests and/or prescription by a licensed veterinarian for any reason, treatment of birds, response to notified symptoms, disease risk factors, or mortality; (3) Therapeutics: Use of drugs after confirmation with laboratory tests and recommendation by licensed veterinarian; and (4) No usage: where the particular drug has not been used within the period covered by this study. All these include extra label drugs as categorized by FARAD under 21 Code of Federal Regulations (CFR) 530 [15]. 

### 2.3. Bacterial Isolates

Biological samples were collected from 60 locations/sites from the six identified stages simultaneously with the questionnaires. The purpose of biological sampling was to determine the prevalence of *Salmonella* in the NBPVC (Figure 1). Identification and isolation of *Salmonella* organisms were conducted using approved protocols as described in Chapter 6, Section 6.2.4. of the Modified ISO 6579. Specifically, a total of 1135 samples were obtained from broiler production inputs, products, and environments along the targeted stages and sites of the NBPVC (Table 1). A total of 507 *Salmonella* isolates were confirmed by *invA* identification PCR as describe in Chapter 6, Section 6.2.6 of the Modified ISO 6579 (Figure 1) [11]. The *Salmonella* bacterial isolates obtained from this study were used in a follow-up study (Figure 1) [11].

### 2.4. Antimicrobial Susceptibility Testing (AST)

Using the ISO/TS 16782:2016 protocol, antimicrobial susceptibility testing (AST) was conducted on the 507 *Salmonella* isolates using Kirby–Bauer disk diffusion method [14]. A total of 18 antimicrobial drugs commonly used in humans in Nigeria, recognizing the antimicrobials listed by the WHO as critically important antimicrobials for humans, were included in the AST assessment [13]. The included drugs were AMC = amoxicillin clavulanic (30 µg), AMP = ampicillin (10 µg), CIP = ciprofloxacin (5 µg), CN = gentamycin (120 µg), CRO = ceftriaxone (30 µg), CT = colistin (10 µg), DO = doxycycline (30 µg), E = erythromycin (15 µg), ENR = enrofloxacin (5 µg), FFC = florfenicol (30 µg), N = neomycin (30 µg), NOR = norfloxacin (10 µg), P = penicillin G (10 µg), PEF = perfloxacin (5 µg), S = sreptomycin (300 µg), SXT = co-trimoxazole (Trimethoprim-Sulphamethoxazole) (25 µg of 1.25/23.75 μg), TE = tetracycline (30 µg), and UB = flumequine (30 µg) (Oxoid, UK) (Table 2).

Susceptibility was determined by preparing an inoculums suspension for each isolate on Tryptose blood agar (CM0233B) and adjusting the inoculum to a McFarland standard of 0.5 for turbidity of bacterial suspensions. Every batch of the inoculums was used immediately or within 15 min of preparation and streaked on agar plates. Antibiotic containing disks were applied firmly on the agar surface within 15 min of inoculation of the plates. Plates were incubated at 35 ± 1 °C for 16–20 h. After incubation, inhibition zones were measured to the nearest millimeter with a ruler.

### 2.5. Data Collection and Analysis

Supportive data on the samples were captured, recorded, and analyzed using Microsoft Excel 2007^®^. Descriptive statistics was used to describe the responses to each category of antimicrobial used and for resistance profiling. Frequency of each category was estimated from the total responses. FARAD was used as a standard to interpret and capture the responses on antibiotic usage [15]. Clinical and Laboratory Standard Institute (CLSI 2018 M100-28) was used for establishing breakpoints for the AST [16]. Isolates were classified as resistant or susceptible using resistance breakpoints as reported by CLSI [16]. The frequency of *Salmonella* isolates resistant to antibiotics was reported as a percentage of the total number of *Salmonella* isolates resistant to an antibiotic against which the pathogens were tested for susceptibility. Further analysis was then done with Microsoft Excel 2007^®^ using simple descriptive statistics, pivot tables, and charts. 

**Figure 2:**
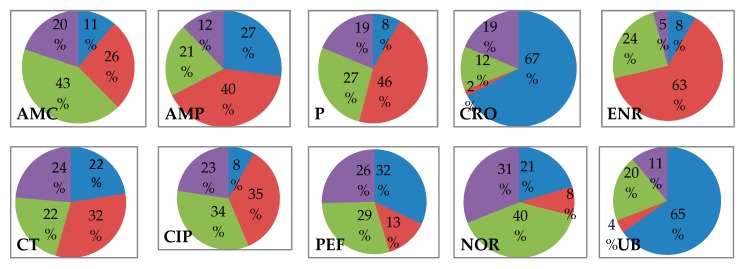

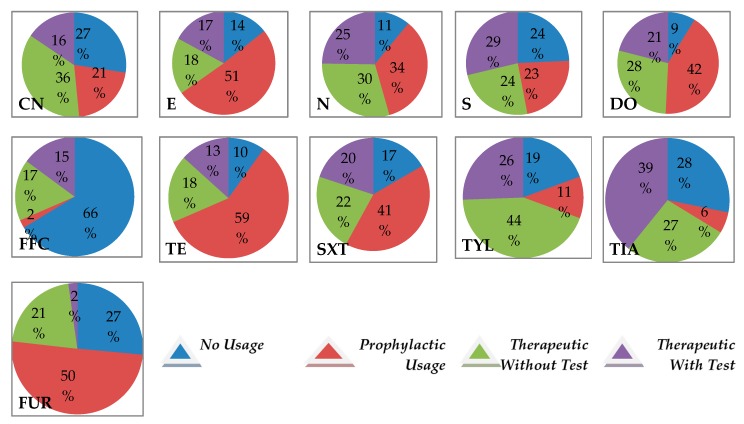
Profiling of purposes of antimicrobial drug administration in the Nigeria broiler production chain Note: AMC = Amoxicillin clavulanic, AMP = Ampicillin, CIP = Ciprofloxacin, CN = Gentamycin, CRO = Ceftriaxone, CT = Colistin, DO = Doxycycline, E = Erythromycin, ENR = Enrofloxacin, FFC = Florfenicol, FUR = Furazolidone, N = Neomycin 30, NOR = Norfloxacin, P = Penicillin G, PEF = Perfloxacin, S = Streptomycin, SXT = Co-trimoxazole (Trimethoprim-Sulphamethoxazole), TE = Tetracycline, TIA = Tiamulin, TYL = Tylosine, UB = Flumequine.

The level of resistance was then analyzed further by categorization for interpretation as described earlier [3]. The sum of percentage of resistance and intermediate level of each antibiotic for AST was regarded as resistant. The pattern of resistance profiling was categorized (Figure 2) as described in a previous study [3]. If the resistance level was less than or up to one (≤1%), the sample was categorized as “no resistance” pattern; between 1 and 25% (>1, ≤25%) was categorized as “very low resistance”; where it was greater than 25 but less than or equal to 50% (>25, ≤50%), it was categorized as “low resistance”; where it was greater than 50 but less than or equal to 75% (>50, ≤75%), it was categorized as “high resistance”; and any value greater than 75% (>75%) was categorized as “very high resistance pattern” (Table 2) [3]. 

### 2.6. Ethical Consideration

The protocols and procedures employed in the study were ethically reviewed and approved prior to the commencement of the study. Approval was obtained from the Animal Ethic Committee of University of Pretoria, South Africa (V062-15 on 01 August 2015) and the Ministry of Health, Oyo State of Nigeria (ref: AD13/479/433). This work was also part of a bioproject registered with the NCBI website, Submission ID, and BioProject ID and accession numbers were SUB4197061, PRJNA477925 and SRP151337, respectively. In addition, the consent for this research was sought from the Poultry Association of Nigeria through the Association Chapter in Oyo State; the same organization encouraged stakeholders’ participation in the study. However, none of the individuals or organizations influenced the study design or implementation of the project.

## 3. Results

### 3.1. Antimicrobial Drug Administration and Usage

Based on the overall patterns of intended use of antibiotics, over 80% (145) of the 181 responses used antibiotics intentionally without veterinarian prescription or laboratory confirmation including the use as extra label antibiotics (Table 1). Stage-specific differences exist as this observed indiscriminate application of antimicrobials without veterinary input (prescription) was highest in the LBMs (100%), hatcheries (87%), grow-out farms (75%), and breeder farms (67%). Similarly, the LBMs (100%) had the highest prevalence rate of *Salmonella* isolates per location, while for the other sampled locations, results indicated the following, hatcheries = 57%, breeder farms = 56%, grow-out farms = 55%, abattoirs = 33%, and retail outlets = 25% (Table 1). 

Based on the antimicrobial drugs sampled, the most abused drugs in the Nigeria broiler production value chain (NBPVC) using the FARAD specification were enrofloxacin and tetracycline. Twelve (12) out of the 21 drugs sampled had high rates of indiscriminate administration without laboratory test or veterinary prescription; these include amoxicillin-clavulanic, ampicillin, ciprofloxacin, colistin, doxycycline, erythromycin, gentamycin, neomycin, penicillin, cotrimoxazole (trimethoprim-sulphamethoxazole), tylosine, and furaltadone (Table 2, Figure 2, Supplementary Materials Table S1). Antimicrobial drugs that are not commonly used and not arbitrarily administered in the NBPVC are few and include ceftriaxone, florfenicol, flumequine, and tiamulin.

### 3.2. Antimicrobial Resistance of Salmonella Isolates Originating from NBPVC

**Figure 3:**
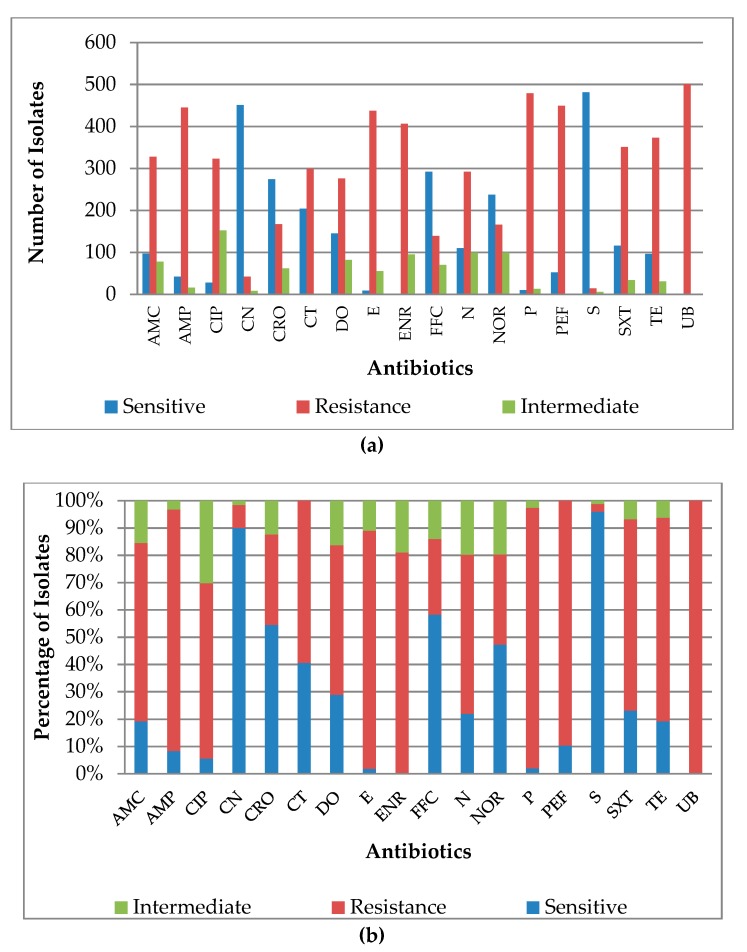
Resistance profile of 507 *Salmonella* isolates from the Nigeria broiler production value chain. (**a**) Distribution of resistance of 507 *Salmonella* isolates from the Nigeria broiler production value chain to each antibiotic; (**b**) Resistance profile of 507 *Salmonella* isolates from the Nigeria broiler production value chain to each antimicrobial. Note: AMC = Amoxicillin clavulanic 30 µg, AMP = Ampicillin 10 µg, CIP = Ciprofloxacin = 5 µg, CN = Gentamycin 120 µg, CRO = Ceftriaxone 30 µg, CT = Colistin 10 µg, DO = Doxycycline 30 µg, E = Erythromycin 15 µg, ENR = Enrofloxacin 5 µg, FFC = Florfenicol 30 µg, N = Neomycin 30 µg, NOR = Norfloxacin 10 µg, P = Penicillin G 10 µg, PEF = Perfloxacin 5µg, S = Streptomycin 300 µg, SXT = Co-trimoxazole (Trimethoprim-Sulphamethoxazole) 25 µg, TE = Tetracycline 30 µg, UB = Flumequine 30 µg.

There was no single antibiotic tested in this research for which the *Salmonella* isolate studied had 100% susceptibility (Figure 3a,b). The highest resistance was observed against flumequine (100%), penicillin (95.4%), perfloxacin (89.6%), ampicillin (88.5%), and enrofloxacin (81%). AMR patterns for the 18 tested antibiotics indicated that 11/18 showed very high levels of resistance for the *Salmonella* isolates tested, including amoxicillin-clavulanic, ampicillin, ciprofloxacin, enrofloxacin, erythromycin, flumequine, neomycin, penicillin, perfloxacin, sulfonamides, and tetracycline. Three of the antibiotics (colistin sulphate, doxycycline, and norfloxacin) revealed high resistance patterns. However, ceftriaxone and florfenicol displayed low resistance patterns, while gentamicin and streptomycin revealed very low resistance patterns (Table 2, Figure 3a,b). These latter antibiotics appear to be the most active against the *Salmonella* isolates in this work.

Comparatively, the trends of use or antimicrobial drug administration closely match with the pattern of antimicrobial resistance except for gentamycin (Table 2, Figure 2, Figure 3a,b). The Salmonella isolates were observed to be highly resistant to most of the drugs that were prophylactically applied and those used for the routine treatment of broilers without testing (Table 2, Figure 2, Figure 3a,b).

Observations on the classes and generations of antimicrobial drugs studied revealed a pattern where the high administration of newer generations of an antibiotic was reflected in high resistance of older generations of the same class such as was seen in enrofloxacin (second generation) and flumequine (first generation), both quinolones. The indiscriminate use of enrofloxacin was very high but indiscriminate use of flumequine was very low, however, both posed very high antimicrobial resistance (81% and 100%, respectively). This pattern was reflected in all classes of antibiotics studied, suggesting a link between antimicrobial usage patterns and AMR development (Table 2, Figure 2, Figure 3a,b).

## 4. Discussion

In this study, evidence of antimicrobial abuse and resistance have been shown; first, it appears that there are no alignments between the dependence on and extensive administration of antimicrobials and the prevalence of *Salmonella* spp. in the NBPVC. However, it should be understood that *Salmonella* infection may occur as a result of many factors, including poor farm biosecurity, bio-importation to the farm from the poultry’s origin, overwhelming endemicity in the farm environment, directly due to farm-level recurrent infection, and probably indirectly due to contamination along the broiler value chain, among others. There was an overall *Salmonella* prevalence of 55% and 23% based on locations and from samples, whereas over 80% of the farmers utilize and abuse antimicrobial drugs as preventive or therapeutic drugs without laboratory diagnostics and veterinary prescriptions. This attitude would accentuate the level of single or multi drug (antimicrobial) resistance situations in the Nigerian broiler industry, especially in situations where compounded drugs and extra-label human drugs are also accessible for use [17,18,19]. It is therefore not surprising that most of the *Salmonella* isolates obtained in this study were highly resistant to the commonly accessible antimicrobials in the market. Previous studies have also indicated other reasons for this increased level of resistance including the carrier status, which is transferable from breeders especially, and induction by stressors, which lowers immunity and exposes the birds to infectious diseases [20,21,22]. 

The findings of this study suggest that salmonellosis is endemic in a good proportion of poultry farms in Nigeria, and in a situation of poorly applied biosecurity and poor implementation of good farming practices, farm-level infections and contamination, or residues may persist right through the broiler value chain from farm to the slaughter-processing schedules [9,22]. Comparatively, the LBM had the highest isolation (prevalence) rate for *Salmonella* as well as usage of drugs without prescription (Table 1). It should be understood that birds often arrive at the LBM in stressed conditions, and salvage sales are often undertaken by farmers in Nigeria to reduce farm-level losses [22]. Furthermore, live-bird marketers usually do not separate batches of new arrivals but rather add them to the old stocks. In this instance, sick poultry may spread infectious pathogens to clinically healthy birds leading to the need for metaphylactic treatment of the whole batch prior to sale. It is not surprising therefore that the marketers resort to un-prescribed antimicrobial administration. Because such drugs can be passed into the human food chain, it becomes imperative that monitoring (especially for residues and drug administration) of poultry displayed for sale should be carried out routinely by the necessary public health, food, and veterinary authorities routinely at the Nigerian LBMs. The level of isolation at the farms and hatcheries are also worrisome and these are intricately linked to poor biosecurity as well as a lack of progressive monitoring in the broiler value chain with consequent overreliance on antimicrobials to prevent massive disease outbreak. Some other studies have earlier confirmed this assertion [17,22,23]. It is therefore evident that the usage of antimicrobials is unguided and indiscriminate based on the observations from the field; in addition, a close assessment of the antimicrobials in use suggests the proliferation of sub-substandard antibiotics in circulation, under-dosing, over-use, and unfettered access to antimicrobials on the shelf [17,18]. Furthermore, a number of antimicrobials are packaged as fortified com-biotics (mixture of multiple antimicrobial agents, minerals, and vitamins). In some of these drugs, antimicrobials are often included in sub-minimal levels or concentrations far below therapeutic requirements and such drugs serve the interest of growth promotion and production rather than for therapeutics [17,18,19]. 

The hatchery management practices of administering antimicrobial to day-old chicks for the purpose of reducing the risk of transfer of vertically transmitted pathogens also complicates the introduction of antimicrobials into the NBPVC. Though the rationale for this drug administration is unfounded, it has been accepted as the norm in the industry by some hatchery operators. It then follows that farmers may often need an increased level of antimicrobials at grow-out farms to achieve “preventative” or therapeutic purposes. In Nigeria, several grow-out farms consistently administer antimicrobial drugs for most part of the poultry management period from day-old to depopulation, sometimes without withdrawal period, and this practice may lead to subclinical salmonellosis in these operations. Whether this situation influences the observed lower recovery rate of *Salmonella* in grow-out farms when compared to breeder farms and hatcheries is not clear, but is not unlikely. Hence, the grow-out farms appear to be big contributors to the AMR patterns in the industry [23]. 

The very high resistance of flumequine (first generation quinolone) despite being the least abused suggests it derived its resistance status from abuse of newer generations of quinolones such as enrofloxacin and perfloxacin (second generation). A similar trend was also recorded in other classes of antimicrobials. There was also very high resistance to some antimicrobials such as penicillin, ampicillin, and erythromycin that used to be readily abused in the LBM without observing the withdrawal periods. Generally, most stakeholders and actors in the Nigeria broiler industry, particularly in the small to medium-scale farms, do not care about observance of withdrawal periods for antimicrobials. A reorganization of this sector becomes necessary, and medicines must clearly stipulate withdrawal periods while adherence must be enforced by government veterinary officers. The industry can similarly push for adherence in this regard in order to reduce the incidence of AMR in the broiler industry. Due to the fact that certain groups and classes of antimicrobials displayed a higher level of resistance compared to others, the drug authorities will need to regularly review access to these antimicrobials to see whether the laws and regulations targeted to their use are in tandem with the realities in the field.

## 5. Conclusions

Evidence of overreliance on and indiscriminate use of antimicrobial drugs have been shown in this study, and prevalence of multidrug resistant *Salmonella* has been revealed in the NBPVC. There is a need to use international standards like FARAD for monitoring, evaluation, and control of the antimicrobial usage and administration in food animals in Nigeria. Professionals must promote good antimicrobial stewardship, as is consistent with globally acceptable standards. Since most of the antimicrobials the *Salmonella* spp. were resistant to in this study are on the WHO List of Essential Antimicrobials and are listed in the OIE Terrestrial Animal Health Code, it necessitates the need for a comprehensive evaluation of the situation of AMR in humans in Nigeria. A carefully planned, multi-sectoral, surveillance plan can be adopted for research and diagnostic purposes in various aspect of AMR in Nigeria. There is a need for standardization in drug administration in all food animals using stewardship and One Health benchmarks. The promotion of good farming practices and antimicrobial stewardship should become the focus of the government and all stakeholders. It is necessary to register and monitor broiler production farms using the value chain approach. 

## 6. Limitations

A pairwise correlation analysis was not conducted for the antimicrobials to compare if a high prevalence of one antimicrobial influences the other, or whether the administration of one antimicrobial influences its resistance pattern. This was due to the difficulties encountered to conduct independent verification of antimicrobial use data provided by the farmers.

## Figures and Tables

**Table 1:** Demography of antimicrobial resistance profiling of *Salmonella* spp. at the production stages along the broiler value chain in Nigeria.

ProductionStages	Location Sampled for AU (n)	AU Samples per Location (n)	AU Responses per Stage (n)	AU Without Lab Test (n (%)	*Salm.* Sampling Locations per Stage (n)	Samples per Stage for *Salm.*Isolation (n)	*Salm.* Prevalence Rates (n) (%)		*Salm.* Isolates for Resistance Profiling (n)
							Based on Locations	Based on Samples	
Breeder	45	1	45	30 (66.7%)	16	332	56.25%	22.29%	146
Hatchery	15	1	15	13 (86.7%)	7	144	57.13%	32.64%	102
Grow-out	76	1	76	57 (75.0%)	22	373	54.55%	18.77%	111
Abattoir	ND	ND	ND	ND	6	151	33.33%	12.58%	46
LBM	15	3	45	45 (100%)	5	79	100%	49.37%	65
Retail	ND	ND	ND	ND	4	56	25.00%	21.43%	37
Total	151	NA	181	145(80.1%)	60	1135	55.00%	23.00%	507

Note: AU = Antimicrobial usage; NA = Not applicable; ND = Not done; lab. = laboratory; Stage = production stage; *Salm.* = *Salmonella*; n = number; *Salm.* prevalence rates based on locations = Percentage of number of location that had at least one of the samples from the location positive for *Salmonella* detection; *Salm.* prevalence rate based on samples = Percentage of number of *Salmonella* positive samples from the total sample collected per stage. *Salmonella* positivity was based on both biochemical tests and *invA* gene PCR detection. LBM = Live Bird Market.

**Table 2:** Antibiotic usage and antimicrobial susceptibility testing of *Salmonella* isolates along the broiler production chain of Nigeria.

Antibiotic	Class (Generation)	Antibiotic Usage Responses			Antimicrobial Susceptibility Testing Report					
		Prophylactic (%)	Therapeutic Without Test (%)	Total usage Without Test %	Categorization of Usage Without Test	Antim. Conc. (µg)	Resistance (%)	Intermediate (%)	Total AMR %	AMR Categorization
AMC	β –Lactam (4th)	26.5	42.5	69	H	30	65.2	15.5	80.7	VH
AMP	β –Lactam (3th)	40.3	20.4	60.7	H	10	88.5	3.2	91.7	VH
CRO	β –Lactam (3th)	1.7	12.2	13.9	VL	30	33.2	12.3	45.5	L
CIP	Quinolone (2th)	35.4	33.7	69.1	H	5	64.2	30.2	94.4	VH
CT	Polypeptide (1st)	31.9	22	53.9	H	10	59.4	0	59.4	H
DO	Tetracycline (NGC)	42	28.2	70.2	H	30	54.9	16.3	71.2	H
ENR	Quinolone (2th)	63	24.3	87.3	VH	5	81.0	19.0	100	VH
E	Macrolide (NGC)	50.8	17.7	68.5	H	15	87.2	11.0	98.2	VH
FFC	Phenicol (NGC)	2.2	16.6	18.8	VL	30	27.7	14.0	41.7	L
UB	Quinolone (1st)	3.9	19.9	23.8	VL	30	100	0	100	VH
CN	Aminoglycoside (NGC)	25.4	43.6	69	H	120	8.4	1.6	10	VL
N	Aminoglycoside (NGC)	34.6	29.7	64.3	H	30	58.3	19.7	78	VH
NOR	Quinolone (2th)	8.3	39.2	47.5	L	10	33.1	19.7	52.8	H
P	β –Lactam (1st)	45.9	27.1	73	H	10	95.4	2.6	98	VH
PEF	Quinolone (2th)	13.3	29.3	42.6	L	5	89.6	0	89.6	VH
S	Aminoglycoside (NGC)	22.7	24.3	47	L	300	2.8	1.2	4	VL
SXT	Sulfonamides (NGC)	41.4	22.1	63.5	H	25	70.1	6.8	76.9	VH
TE	Tetracycline (NGC)	58.6	18.2	76.8	VH	30	74.6	6.2	80.8	VH
TYL	Macrolides (NGC)	11.1	43.6	54.7	H	ND	ND	ND	ND	ND
TIA	Pleuromutilins (NGC)	5.5	27.1	32.6	L	ND	ND	ND	ND	ND
FUR	Nitrofuran (NGC)	50.3	21	71.3	H	ND	ND	ND	ND	ND

AMC = Amoxicillin clavulanic, AMP = Ampicillin, CIP = Ciprofloxacin, CN = Gentamycin, CRO = Ceftriaxone, CT = Colistin, DO = Doxycycline, E = Erythromycin, ENR = Enrofloxacin, FFC = Florfenicol, FUR = Furazolidone, N = Neomycin 30, NOR = Norfloxacin, P = Penicillin G, PEF = Perfloxacin, S = Streptomycin, SXT = Co-trimoxazole (Trimethoprim-Sulphamethoxazole), TE = Tetracycline, TIA = Tiamulin, TYL = Tylosine, UB = Flumequine, n = number of sample or response for antibiotic usage and number of *Salmonella* isolates for antimicrobial susceptibility testing; % = Percentage of the observed over total for each antibiotics. ND = Not Done. NGC = No generation classification is applicable. VH = Very High, H = High, L = Low, VL = Very Low. Total Usage without test = Prophylactic + Therapeutic without test. Total antimicrobial resistance (AMR) = Resistance + Intermediate. Usage and AMR categorization = Categorization of total usage without test or total AMR into: No (≤1%), Very low (>1, ≤25%), Low (26 ≤ 50%), High (51 ≤ 75%), and Very high (≥75%). Antib conc. = Antibiotic concentration used for antimicrobial resistance testing. µg = microgram.

## Supplementary Materials

The following are available online at https://www.mdpi.com/2079-6382/8/2/75/s1: Figure S1: Flow chart of the methods involved in isolation and identification of *Salmonella* species along the broiler production value chain in Nigeria; Figure S2: Gel image of optimized *invA* gene PCR used for identification of *Salmonella* species isolated from the Nigeria broiler production value chain; Table S1: Data for antibiotic usage and antimicrobial susceptibility testing of *Salmonella* isolates along the broiler production chain of Nigeria.
