# Supplementary material for: Antimicrobial Drug Administration and Antimicrobial Resistance of Salmonella Isolates Originating from the Broiler Production Value Chain in Nigeria
Source: Antibiotics (Basel). 2019 Jun 6;8(2):75. doi: 10.3390/antibiotics8020075 (PMC6627717; doi:10.3390/antibiotics8020075)
Supplement: Supplementary file 1 [file antibiotics-08-00075-s001.zip › Supplemetary file/Suppl. Figure S2.pdf]

- -	M	1	4	6	11	15	23	67	83	++	++	- -	- -	E	89	E

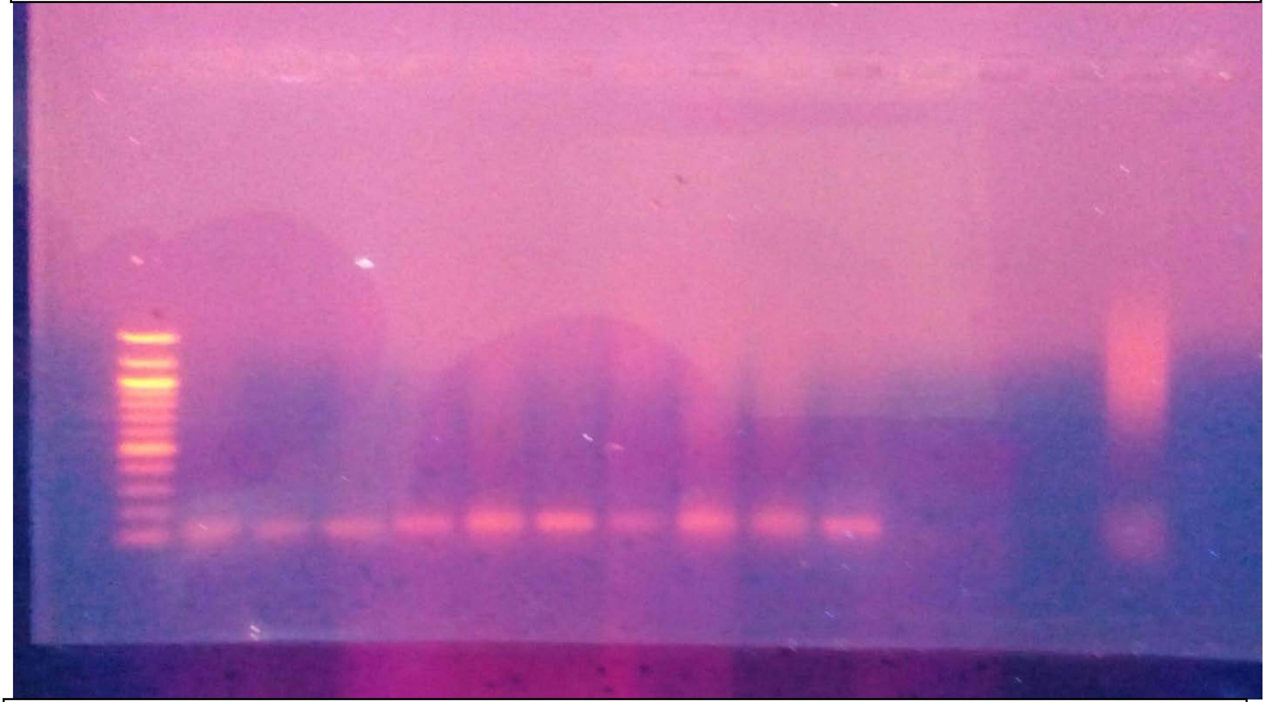

Note: - - = Negative.Control (No DNA). ++ =Positive control (Salmonella Typhimurium ATCC14028). E=Empty. 1, 4, 6, 11, 15, 23, 67, 83, 89 are biochemical confirmed Salmonella isolates. Expected bp= 119bp

Figure S 2: Gel image of optimized invA gene PCR used for identification of Salmonella spp isolated from the Nigeria broiler production value chain
