# Supplementary material for: Antimicrobial Drug Administration and Antimicrobial Resistance of Salmonella Isolates Originating from the Broiler Production Value Chain in Nigeria
Source: Antibiotics (Basel). 2019 Jun 6;8(2):75. doi: 10.3390/antibiotics8020075 (PMC6627717; doi:10.3390/antibiotics8020075)
Supplement: Supplementary file 1 [file antibiotics-08-00075-s001.zip › Supplemetary file/Suppl._Figure_S1.pdf]

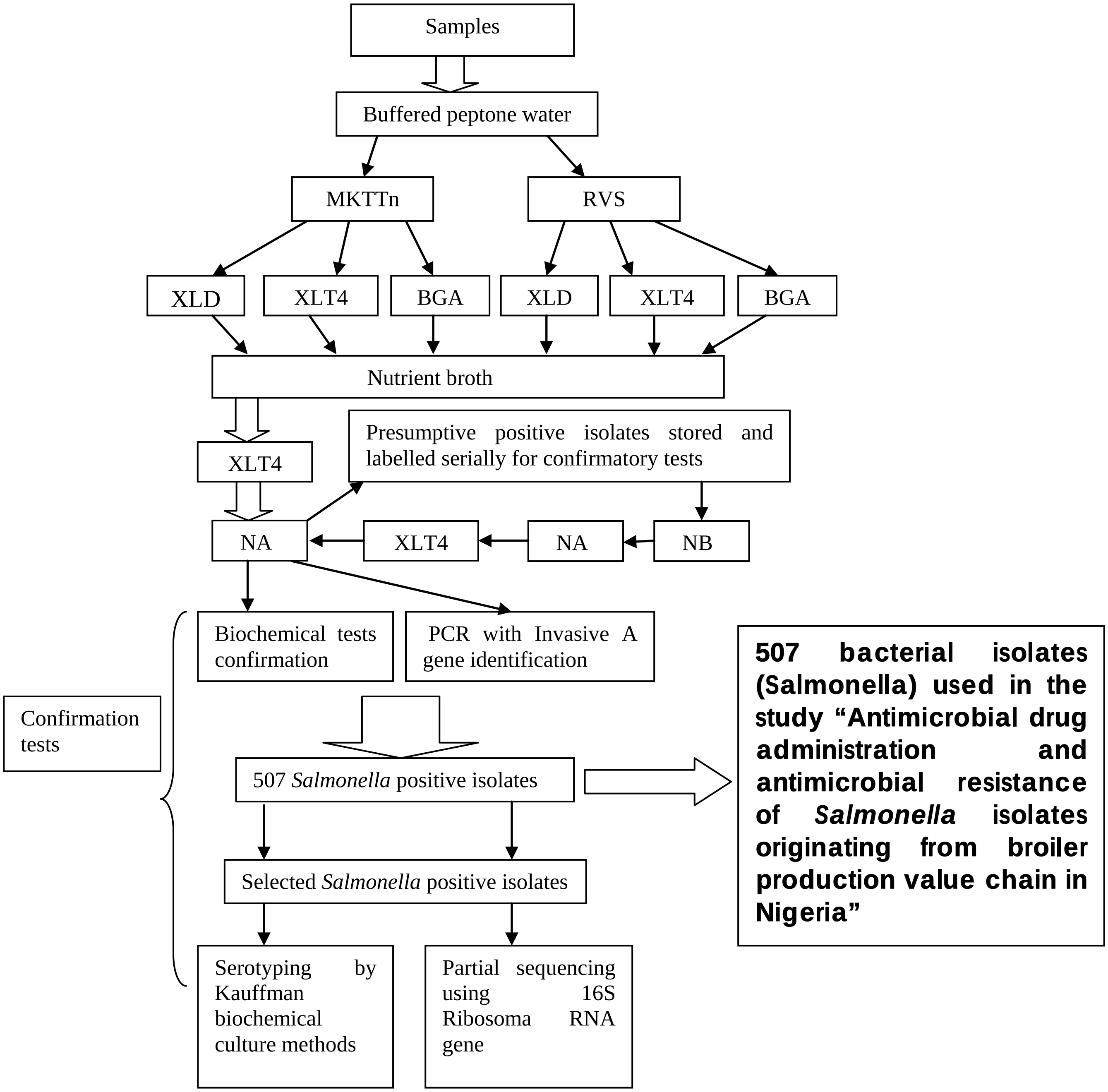

Note: MKTTn= Muller Kauffmann tetrathionate-novobiocin broth. RVS= Rappaport-Vassiliadis soya peptone broth. XLD= Xylose lysine deoxycholate agar. XLT4= Xylose lysine tergitol4. BGA= Brilliant green agar, NA= Nutrient agar. NB= Nutrient broth

Figure S1: Flow chart of the methods involved in isolation and identification of *Salmonella* species along the broiler production value chain in Nigeria
