# Supplementary material for: Antimicrobial Drug Administration and Antimicrobial Resistance of Salmonella Isolates Originating from the Broiler Production Value Chain in Nigeria
Source: Antibiotics (Basel). 2019 Jun 6;8(2):75. doi: 10.3390/antibiotics8020075 (PMC6627717; doi:10.3390/antibiotics8020075)
Supplement: Supplementary file 1 [file antibiotics-08-00075-s001.zip › Supplemetary file/Supplemetary Table.docx]

**Table S 1: Data for antibiotic usage and antimicrobial susceptibility testing of Salmonella isolates along the broiler production chain of Nigeria**

| Antibiotic | | Antibiotic usage responses | | | | Antimicrobial susceptibility testing report | | | |
| --- | --- | --- | --- | --- | --- | --- | --- | --- | --- |
|  | Class  (Generation) | Prophylactic n(%) | Therapeutic without test n (%) | Therapeutic with test n (%) | Non usage n (%) | Antib. Con(µg) | Sensitive n (%) | Intermediate n (%) | Resistance n (%) |
| AMC | β –Lactam (4^th^) | 48 (26.5) | 77 (42.5) | 36 (19.9) | 20 (11.1) | 30 | 97 (19.3) | 78 (15.5) | 328 (65.2) |
| AMP | β –Lactam (3^th^) | 73 (40.3) | 37 (20.4) | 22 (12.2) | 49 (27.1) | 10 | 42 (8.3) | 16 (3.2) | 445 (88.5) |
| CRO | β –Lactam (3^th^) | 3 (1.7) | 22 (12.2) | 34 (18.8) | 122 (67.3) | 30 | 274 (54.5) | 62 (12.3) | 167 (33.2) |
| CIP | Quinolone (2^th^) | 64 (35.4) | 61 (33.7) | 41 (22.7) | 15 (8.3) | 5 | 28 (5.6) | 152 (30.2) | 323 (64.2) |
| CT | Polypeptide (1^st^) | 61 (31.9) | 42 (22.0) | 45 (23.6) | 43 (22.5) | 10 | 204 (40.6) | 0 (0) | 299 (59.4) |
| DO | Tetracycline (NGC) | 76 (42.0) | 51 (28.2) | 38 (21.0) | 16 (8.8) | 30 | 145 (28.8) | 82 (16.3) | 276 (54.9) |
| ENR | Quinolone (2^th^) | 114 (63.0) | 44 (24.3) | 8 (4.4) | 15 (8.3) | 5 | 0 (0) | 95 (19.0) | 406 (81.0) |
| E | Macrolide (NGC) | 92 (50.8) | 32 (17.7) | 31 (17.1) | 26 (14.4) | 15 | 9 (1.8) | 55 (11.0) | 437 (87.2) |
| FFC | Phenicol (NGC) | 4 (2.2) | 30 (16.6) | 27 (14.9) | 120 (66.3) | 30 | 292 (58.3) | 70 (14.0) | 139 (27.7 |
| UB | Quinolone (1^st^) | 7 (3.9) | 36 (19.9) | 20 (11.1) | 118 (65.2) | 30 | 0 (0) | 0 (0) | 501 (100) |
| CN | Aminoglycoside (NGC) | 46 (25.4) | 79 (43.6) | 34 (18.8) | 60 (33.2) | 120 | 451 (90.0) | 8 (1.6) | 42 (8.4) |
| N | Aminoglycoside (NGC) | 63 (34.4) | 54 (29.5) | 45 (24.6) | 20 (10.9) | 30 | 110 (22.0) | 99 (19.7) | 292 (58.3) |
| NOR | Quinolone (2^th^) | 15 (8.3) | 71 (39.2) | 55 (30.4) | 40 (20.1) | 10 | 237 (47.2) | 99 (19.7) | 166 (33.1) |
| P | β –Lactam (1^st^) | 83 (45.9) | 49 (27.1) | 34 (18.8) | 15 (8.3) | 10 | 10 (2.0) | 13 (2.6) | 479 (95.4) |
| PEF | Quinolone (2^th^) | 24 (13.3) | 53 (29.3) | 46 (25.4) | 58 (32.00 | 5 | 52 (10.4) | 0 (0) | 449 (89.6) |
| S | Aminoglycoside (NGC) | 41 (22.7) | 44 (24.3) | 52 (28.7) | 44 (24.3) | 300 | 481 ((96.0) | 6 (1.2) | 14 (2.8) |
| SXT | Sulfonamides (NGC) | 75 (41.4) | 40 (22.1) | 36 (19.9) | 30 (16.6) | 25 | 116 (23.2) | 34 (6.8) | 351 (70.1) |
| TE | Tetracycline (NGC) | 106 (58.6) | 33 (18.2) | 24 (13.3) | 18 (9.9) | 30 | 96 (19.2) | 31 (6.2) | 373 (74.6) |
| TYL | Macrolides (NGC) | 20 (11.1) | 79 (43.6) | 47 (25.4) | 35 (19.3) | ND | ND | ND | ND |
| TIA | Pleuromutilins (NGC) | 10 (5.5) | 49 (27.1) | 71 (39.2) | 51 (28.2) | ND | ND | ND | ND |
| FUR | Nitrofuran (NGC) | 91 (50.3) | 38 (21.0) | 4 (2.2) | 48 (26.5) | ND | ND | ND | ND |

AMC= Amoxicillin clavulanic, AMP= Ampicillin, CIP= Ciprofloxacin, CN= Gentamycin, CRO= Ceftriaxone, CT= Colistin, DO= Doxycycline, E= Erythromycin, ENR= Enrofloxacin, FFC= Florfenicol, FUR= Furazolidone, N= Neomycin 30, NOR= Norfloxacin, P= Penicillin G, PEF= Perfloxacin, S= Streptomycin, SXT= Co-trimoxazole (Trimethoprim-Sulphamethoxazole), TE= Tetracycline, TIA= Tiamulin, TYL= Tylosine, UB= Flumequine, n= number of sample or response for antibiotic usage and number of *Salmonella* isolates for Antibiotic sensitivity testing; %= Percentage of the observed over total for each antibiotics. Antib conc.= Antibiotic concentration used for antimicrobial resistance testing. µg= microgram.
